# Determinants of blood lead and cadmium levels in sanitary landfill workers: a cross-sectional study in Greece

**DOI:** 10.3389/fpubh.2026.1812981

**Published:** 2026-05-04

**Authors:** Argyri Kareli, Viktoria Maria Amoiridou, Theodoros Amoiridis, Christos Kontogiorgis, Theodoros C. Constantinidis, Evangelia Nena

**Affiliations:** 1 Laboratory of Hygiene and Environmental Protection, Department of Medicine, Democritus University of Thrace, Alexandroupolis, Greece; 2 Laboratory of Social Medicine, Department of Medicine, Democritus University of Thrace, Alexandroupolis, Greece

**Keywords:** cadmium, Greece, heavy metal, landfill, lead

## Abstract

**Background-aim:**

Landfill workers face a range of occupational exposures that can adversely affect their health and overall quality of life. The aim of this study was to assess cadmium and lead concentrations in the blood of workers employed at sanitary landfill sites, while documenting additional health indicators. Another objective was to explore potential associations between exposure levels and demographic factors, occupational history, working conditions.

**Methods:**

A cross-sectional study was conducted among employees working at four landfill sites in Northern Greece. Participants completed a questionnaire and underwent clinical examination. Blood samples were collected for hematological, biochemical analyses, and the determination of lead and cadmium concentrations. Statistical analyses were performed to identify differences in the examined parameters across occupational exposure groups and to identify predictors of lead and cadmium levels.

**Results:**

A total of 147 employees were recruited (76.9% males, n = 113), with a mean age of 48 ± 7.6 years. Blood lead levels were significantly higher in males compared to females (2.26 μg/dL vs. 1.40 μg/dL, *p* = 0.001), while no sex differences were observed for cadmium (*p* = 0.438). Increasing age was associated with higher lead levels (*p* = 0.001), but not with cadmium (*p* = 0.382). Cadmium levels increased with higher BMI, whereas lead levels decreased (normal weight: 2.24 ± 1.67 μg/dL and 1.25 ± 1.33 μg/L; overweight: 2.05 ± 1.33 μg/dL and 1.41 ± 1.97 μg/L; obese: 1.88 ± 1.15 μg/dL and 1.45 ± 1.89 μg/L, respectively). Smoking was a significant determinant of both metals, with higher levels observed among smokers (lead: 1.61 ± 0.98 μg/dL; cadmium: 0.50 ± 0.71 μg/L). Significant predictors of cadmium and lead levels included smoking, duration of employment, work position, occupational site and use of personal protective equipment (PPE). Highly exposed workers had significantly higher cadmium (2.12 ± 2.38 μg/L) and lead levels (2.69 ± 1.61 μg/dL) compared to moderate and low exposure groups (*p* ≤ 0.016).

**Conclusion:**

Blood cadmium and lead levels among landfill workers were associated with occupational exposure intensity and individual factors such as age, BMI, and smoking. Although overall concentrations were relatively low, cumulative exposure influenced by both workplace conditions and lifestyle factors is evident. These findings underscore the need for ongoing occupational health monitoring and targeted preventive measures to reduce exposure and protect worker health.

## 1 Introduction

Sanitary landfills are engineered facilities composed of sealed underground cells designed for waste disposal. At the base of these disposal areas, leachate is generated as water percolates through waste. This leachate is collected and treated through processes such as recycling, evaporation, spraying, and controlled discharge. It contains a wide range of chemical compounds, including heavy metals such as lead (Pb) and cadmium (Cd), which pose significant risks to human health (1–6).

Previous studies have demonstrated that workers involved in waste management activities may experience increased exposure to heavy metals. For example, workers engaged in precipitate cleaning have been shown to exhibit elevated blood lead levels (1), while recycling workers are also at increased risk due to direct contact with waste materials (2, 7, 8). The use of personal protective equipment (PPE) has been associated with reduced exposure levels (1), highlighting the importance of preventive measures. However, available evidence remains heterogeneous, and findings are often context-specific. Studies conducted in Thailand have examined blood lead and cadmium levels in relation to PPE use and hygiene practices (9), while environmental studies in Greece have documented heavy metal contamination in landfill soils (5). Nevertheless, data on internal exposure levels among landfill workers, particularly in European settings, remain limited.

A substantial body of evidence indicates that heavy metal contamination of ecosystems is largely attributable to human activities (1, 10–12). Once released into the environment, these metals can disrupt normal biological functions, cause irreversible damage to critical organs, and, in severe cases, result in mortality (7, 13–15). Exposure to heavy metals has been associated with chronic diseases such as type II diabetes, Alzheimer’s disease, and hypertension (16–18). Human exposure to soil-derived heavy metals occurs primarily through ingestion, inhalation, and dermal contact (4–6). Therefore, the implementation of appropriate protective measures—such as the use of masks, gloves, and protective work clothing—is essential for workers at risk of occupational exposure.

Despite these concerns, there is limited evidence on biomonitoring of heavy metal exposure among landfill workers, particularly in Southern Europe, where occupational conditions and waste management practices may differ from those reported elsewhere. Furthermore, few studies have simultaneously examined the combined influence of occupational exposure, workplace conditions, and individual lifestyle factors on internal metal burden.

The objective of the present study was to assess blood levels of Pb and Cd among landfill workers in Northern Greece and to investigate their association with demographic characteristics, occupational history, working conditions, and lifestyle factors. Pb and Cd were selected as target pollutants due to their widespread presence in municipal solid waste, persistence in the environment, bioaccumulative properties, and well-documented toxic effects on multiple organ systems. Additionally, both metals are widely used as biomarkers in occupational biomonitoring, enabling comparison with existing literature. A secondary aim was to evaluate general health indicators, including hematological and biochemical parameters, as to explore potential correlations with the concentrations of Pb and Cd.

## 2 Materials and methods

The study had a cross-sectional design and included employees from various job positions across four sanitary landfill sites in the area surrounding the city of Thessaloniki, in Northern Greece.

Inclusion criteria were the following: being employed for at least 1 year and consent to participate. No exclusion criteria were applied, except for refusal to participate.

Participants were recruited over an 18-month period. All eligible workers were invited to participate and were scheduled for evaluation at the Occupational Medicine Department. Upon attendance, participants were informed about the study procedures and provided written informed consent prior to enrollment.

Face-to-face interviews were conducted, followed by clinical examination at the Occupational Medicine Department. A structured questionnaire was administered to collect information from the participating workers, covering anthropometric characteristics, family status, educational level, lifestyle habits (e.g., smoking and alcohol consumption), individual medical history, occupational history, family medical history, and use of personal protective equipment. The questionnaire was developed based on commonly used items in occupational health research to capture demographic, occupational, and lifestyle information; however, formal validation and reliability testing were not performed.

Moreover, venous blood samples were collected from each participant, and three 5 mL aliquots were prepared and stored in acid-cleaned plastic tubes. These samples were used for hematological and biochemical analyses, as well as for the determination of lead (Pb) and cadmium (Cd) concentrations. Metal analysis was performed using Inductively Coupled Plasma Mass Spectrometry (ICP-MS) (Analytik Jena, PlasmaQuant MS, Aspect MS software v3.3.4.7), a highly sensitive technique for trace element detection.

For trace metal analysis, blood samples were collected in metal-free tubes to prevent contamination and subsequently diluted (1:10 or 1:20) with a solution containing NH₄OH, Triton X-100, and EDTA to ensure hemolysis and stabilization of metals. Internal standards (rhodium and iridium) were applied to correct for instrumental variability, and analyses were conducted using a helium collision gas system. Calibration was performed on a daily basis, including calibration blanks prepared with the same diluent as the samples. Quality control was ensured using certified reference materials (Recipe Clin Chek) at three concentration levels.

The limits of detection (LOD) ranged from 0.0001 to 0.0005 μg/L for Pb and from 0.0001 to 0.0008 μg/L for Cd, while the limits of quantification (LOQ) were 0.38 μg/L for Pb and 0.30 μg/L for Cd.

Participants were categorized into four groups according to their worksite and corresponding exposure level: (1) workers employed at an active landfill site (direct exposure); (2) workers at a recycling material management facility (direct exposure); (3) workers at an inactive landfill site (former waste disposal areas) (indirect exposure); and (4) workers at a rehabilitated/ environmental park site, i.e., a former landfill area that has been inactive for more than 35 years (indirect exposure).

Exposure groups were formed based on job title, specific work tasks, and worksite characteristics, as defined in the Written Occupational Risk Assessments conducted in accordance with national legislation (Law 3850/2010), which systematically evaluate occupational hazards and exposure levels. This classification, derived from standardized occupational risk assessment procedures, was used as a proxy for relative exposure intensity and is consistent with established occupational health risk management practices, supporting its validity for exposure stratification in the present study.

Table 1 provides an overview of employees’ job tasks and duties, along with the corresponding levels of exposure across the four groups.

**Table 1:** Characteristics of the work positions and settings in the four examined sites.

Sites	Work positions	Setting	Related duties/task description	Level of exposure
1. Active landfill area	Machine operators Drivers Signalmen	Waste unloading process, burial operations, waste disposal, waste coverall, leachate management system	Landfilling, spreading, compacting and covering the waste, according to the program and the instructions of supervisor. Driving vehicles to the area, Various other tasks (e.g., Water spraying on the roads, good maintenance of the vehicles)	Direct/High
	General Labor (including maintenance, cleaning personnel)		Watering, assisting operators in cleaning machinery, assisting in guiding vehicles to disposal sites, transportation of materials and tools. The maintainers are responsible for the maintenance and repair (painting, etc.) of the landfill administration building and its other facilities.	Direct/Moderate
	Supervisors Administrative personnel		Monitors, controls and intervenes in the waste disposal process in accordance with the site development plan. Specifically: Personal supervision of the operation of the unit’s services.	Direct/Low
2. Recycling management center	Recycling Workers	Recycling material management center	Sorting and separate items	Direct/High
3. Inactive landfill area	Administrative personnel Workers with general duties	Inactive landfill area, tree planting, and the general care on of the area	Operations manager, Tree planting, gardening and cleaning	Indirect/low
4. Environmental/rehabilitated parks (former landfill areas)	Administrative personnel Workers with general duties	Environmental Parks (Former Landfill Areas) Stopped operating 35 years ago. Planted with trees- parks in urban tissue, open to the public.	The area Operations Manager, Cleaning, tree planting, watering, mowing, gardening	Indirect/Low

### 2.1 Ethical considerations and approvals

Permission to conduct the study was granted by the Regional Association of Solid Waste Management Bodies of Central Macedonia, Greece (protocol no. 8443/24-08-2016; meeting no. 27/2016; decision no. 633). Ethical approval was subsequently obtained from the Ethics Committee of the Democritus University of Thrace (protocol no. 44844/75; approval date: 24 May, 2017). The study was conducted in accordance with applicable ethical standards and regulations. Informed consent was obtained from all participants prior to their participation in the study.

### 2.2 Statistical analysis

Data were entered and managed using Microsoft Excel and statistical analyses were performed with IBM SPSS Statistics for Windows (Version 22.0). Continuous variables are presented as mean ± standard deviation and categorical variables as frequencies and percentages. Normality and homogeneity of variances were assessed using the Kolmogorov–Smirnov and Levene tests, respectively. Group comparisons were performed using independent samples t-tests or one-way ANOVA. Welch’s ANOVA with Games-Howell *post hoc* tests was applied when homogeneity of variance was violated. Nonparametric tests were used, where appropriate. Correlations between categorical variables were examined using the chi-squared test. Bivariate analyses were performed to examine associations between blood lead and cadmium concentrations and demographic characteristics, behavioral risk factors (smoking status and alcohol consumption), and occupational parameters (workplace, job category, and tenure). Variables that showed statistical significance were included in hierarchical linear regression models to identify independent predictors of metal concentrations. Predictors were entered in sequential blocks (demographic, behavioral, and occupational factors) to assess their contribution to the explained variance. Model assumptions (linearity, normality, homoscedasticity, and multicollinearity) were assessed using standard diagnostic criteria. Statistical significance was set at 0.05.

## 3 Results

### 3.1 General characteristics of the participants

The total sample included 147 workers. (76.9% males, n = 113), with a mean age of 48 ± 7.6 years. Of them, 44.2% (n = 65) were overweight, and 26.5% (n = 39) obese. The majority (54.4%) were active smokers, 25.2% were former smokers, and 20.4% were non-smokers. More detailed information is presented in Table 2.

**Table 2:** General characteristics (demographic, anthropometric), behavioral risk factors (smoking habits and alcohol use), occupational characteristics and working conditions of participants.

Characteristic	Category	*n* (%)
Age	≤ 43	37 (25.2%)
	44–53	78 (53.1%)
	≥ 54	32 (21.8%)
BMI status	Normal weight	43 (29.3%)
	Overweight	65 (44.2%)
	Obese	39 (26.5%)
Smoking	Non-smokers	30 (20.4%)
	Ex-smokers	37 (25.2%)
	Smokers	80 (54.4%)
Age of smoking onset (†)	≤ 18 years	45 (56.3%)
	> 18 years	35 (43.7%)
Cigarettes/day (†)	< 20	30 (37.5%)
	≥ 20	50 (62.5%)
Pack-years (††)	Non-smokers	67 (45.6%)
	Light	26 (17.7%)
	Moderate	41 (27.9%)
	Heavy	13 (8.8%)
Alcohol use	No	134 (91.2%)
	Yes	13 (8.8%)
Work position	High exposure	51 (34.7%)
	Moderate exposure	69 (46.9%)
	Low exposure	27 (18.4%)
Occupational site	Active landfill	76 (51.7%)
	Recycling center	19 (12.9%)
	Inactive landfill	33 (22.4%)
	Environmental park	19 (12.9%)
Years of employment	< 10 years	58 (39.5%)
	10–19 years	53 (36.1%)
	≥ 20 years	36 (24.5%)

(†) Data refer to self-reported smokers only.

(††) Pack-years = (Number of cigarettes smoked per day/20) × Years of smoking.

Non-smokers = 0 pack years, Light smokers = 1–20 pack years, Moderate smokers = 20–40 pack years, Heavy smokers ≥ 40 pack years.

### 3.2 Working environment and exposures

As seen in Table 2, participants were employed in four different waste management areas. More specifically, over half of employees (51.7%, n = 76) were employed in an active landfill (Site 1), 19 participants (12.9%) worked in recycling facilities (Recyclable Materials Management Center- Site 2). Moreover, 22.4% (n = 33) were employed in an inactive landfill (Site 3) and 19 (12.9%) in site 4, a rehabilitated/ environmental park (that stopped operating >35 years ago).

Based on exposure levels, 51 employees (34.7%) were classified as highly exposed, including machine operators, drivers, and flagmen; all individuals in this group were male. Sixty-nine employees (46.9%) were moderately exposed, comprising general labor and maintenance personnel. The remaining 27 employees (18.4%) were categorized as having low exposure and included supervisors and administrative staff.

Among the participants, 39.5% (n = 58) had less than 10 years of experience in their current position or specialty, 36.1% (n = 53) had between 10 and 19 years of experience, and 24.5% (n = 36) had more than 20 years of work experience.

### 3.3 Laboratory exams

To assess the overall health status of the participating workers, a set of hematological and biochemical laboratory tests was conducted. The detailed results of the hematological analyses in the total sample and comparison of differences between sites is displayed in Table 3.

**Table 3:** Hematological-biochemical parameters and blood pressure of the participants (in total and by work position).

		Work position				
Parameter *(normal range)*	Total sample	Machine operators–drivers–Flagmen (*n* = 51)	General labor and maintenance personnel (*n* = 69)	Supervisory and administrative personnel (*n* = 27)	Test statistic	*p*-value
	Mean (SD)	Mean (SD)	Mean (SD)	Mean (SD)	F*	
Serum glucose (70–110 mg/dL)	97.39 (16.87)	98.08 (19.66)	97.06 (15.19)	96.92 (15.82)	*F*(2.144) = 0.06	*p* = 0.937
Creatinine (0.6–1.4 mg/dL)	0.91 (0.17)	0.97 (0.14)	0.88 (0.18)	0.88 (0.16)	*F*(2.144) = 4.04	***p* = 0.020**
Aspartate aminotransferase (AST) (11–38 iu/l)	19.88 (8.41)	20.78 (6.88)	19.97 (10.12)	17.96 (5.69)	*F*(2.144) = 1.00	*p* = 0.370
Alanine aminotransferase (ALT) (11–43 iu/l)	25.39 (14.24)	27.29 (10.75)	24.90 (16.78)	23.07 (12.88)	*F*(2.144) = 0.85	*p* = 0.429
White blood cells (4,000-10,000/ mm^3^)	7254.35 (2021.14)	7436.47 (1659.23)	7193.62 (2126.66)	7065.55 (2388.32)	*F*(2.144) = 0.35	*p* = 0.703
Hemoglobin (12–16 g/100 mL)	14.37 (1.42)	15.04 (1.17)	14.13 (1.40)	13.73 (1.46)	*F*(2.144) = 10.60	***p* < 0.001**
Hematocrit (36–46%)	42.60 (3.69)	44.56 (3.13)	41.76 (3.47)	41.02 (3.79)	*F*(2.144) = 13.35	***p* < 0.001**
Platelets (140,000-440,000 /mm^3^)	241299.32 (53951.71)	231019.61 (45146.65)	243130.43 (57690.33)	256037.04 (57265.56)	*F*(2.144) = 2.00	*p* = 0.139
Neutrophils (40–75%)	56.62 (6.92)	56.43 (6.96)	56.38 (7.06)	57.59 (6.60)	*F*(2.144) = 0.33	*p* = 0.723
Lymphocytes (20–45%)	32.98 (6.29)	32.88 (6.27)	33.48 (6.41)	31.89 (6.12)	*F*(2.144) = 0.62	*p* = 0.537
Monocytes (2–10%)	7.21 (1.84)	7.45 (1.86)	7.15 (1.98)	6.92 (1.36)	*F*(2.144) = 0.80	*p* = 0.450
Eosinophils (1–6%)	2.58 (1.70)	2.69 (1.67)	2.43 (1.59)	2.74 (2.03)	*F*(2.144) = 0.47	*p* = 0.627

Bold Values indicate statistical significance.

### 3.4 Bivariate analyses of laboratory blood tests and cardiovascular parameters, based on participants’ work position

Bivariate analyses were performed to assess differences in laboratory blood parameters across exposure-level groups. As seen in Table 3, significant differences were observed among employees in the three different work positions in creatinine, hemoglobin, and hematocrit levels. Specifically, in the high-exposure group (i.e., operators, drivers, and flagmen) significantly higher hemoglobin and hematocrit values were found compared with general labor and maintenance personnel, as well as supervisory and administrative staff.

It should be also noted, that no statistically significant differences were identified between occupational groups in heart rate, systolic blood pressure, or diastolic blood pressure (all *p* > 0.05), with only minimal variation observed across specialties.

### 3.5 Blood cadmium and lead levels

The analysis showed a statistically significant difference in blood lead levels between males and females, with higher concentrations observed in males (mean 2.26 ± 1.49 μg/dL) compared to females (mean 1.40 ± 0.69 μg/dL), *p* = 0.001. In contrast, no significant sex difference was found for cadmium levels *p* = 0.438.

Increasing age was significantly associated with increased blood lead concentrations (*p* = 0.001), but not with cadmium levels (*p* = 0.382).

Behavioral factors, particularly smoking and alcohol use, were associated with increased blood concentrations of both lead and cadmium. Smokers had significantly higher mean levels of lead and cadmium compared to ex-smokers and non-smokers. When smoking exposure was further classified by pack-years, moderate (20–40 pack-years) and heavy (≥40 pack-years) smokers showed progressively higher blood concentrations of both metals, indicating a clear dose–response relationship [lead: *p* = 0.001; cadmium: *p* < 0.001]. Similarly, participants who smoked at least 20 cigarettes per day had significantly higher lead and cadmium levels than those who smoked fewer cigarettes per day [lead: *p* = 0.011; cadmium, *p* = 0.006].

Alcohol consumption was also associated with increased heavy metal levels. Participants who reported alcohol use had significantly higher blood concentrations of both lead (3.45 vs. 1.93 μg/dL) and cadmium (2.88 vs. 1.23 μg/L) compared with those who did not report alcohol consumption (lead: *p* < 0.001; cadmium: *p* = 0.001). A comprehensive summary of the bivariate analyses examining associations between heavy metal levels and general and behavioral characteristics is presented in Table 4.

**Table 4:** Blood lead and cadmium levels according to general characteristics and behavioral risk factors of the participants (smoking habits and alcohol use).

Characteristic–parameter	Total (*Ν* = 147)	Blood lead level (μg/dL)	Test statistic^*^–*p*-value	Blood cadmium level (μg/L)	Test statistic^*^–*p*-value
	*n* (%)	Mean (SD)		Mean (SD)	
Sex					
Male	113 (76.9%)	2.26 (1.49)	*t*(145) = 3.25	1.44 (1.85)	***t*** (145) = 0.78
Female	34 (23.1%)	1.40 (0.69)	***p* = 0.001**	1.17 (1.52)	*p* = 0.438
Age					
≤ 43	37 (25.2%)	1.50 (0.83)	*F_W_*(3.56.74) = 7.99	1.02 (0.99)	***F*** (2.144) = 0.97
44–53	78 (53.1%)	2.08 (1.40)	***p* = 0.001**	1.49 (2.02)	*p* = 0.382
≥ 54	32 (21.8%)	2.66 (1.66)		1.51 (1.84)	
Educational level					
Primary/secondary school	36 (24.5%)	2.34 (1.51)	*F*(2.144) = 1.29	1.40 (1.75)	*F*(2.144) = 1.23
High school/vocational school	90 (61.2%)	2.02 (1.41)	*p* = 0.278	1.49 (1.93)	*p* = 0.297
University	21 (14.3%)	1.74 (1.05)		0.82 (0.82)	
BMI status					
Normal weight	43 (29.3%)	2.24 (1.67)	*F*(2.144) = 0.66	1.25 (1.33)	*F*(2.144) = 0.15
Overweight	65 (44.2%)	2.05 (1.33)	*p* = 0.518	1.41 (1.97)	*p* = 0.860
Obesity	39 (26.5%)	1.88 (1.15)		1.45 (1.89)	
Alcohol use					
No	134 (91.2%)	1.93 (1.26)	*t*(145) = −3.95	1.23 (1.53)	*t*(145) = −3.30
Yes	13 (8.8%)	3.45 (1.96)	***p* < 0.001**	2.88 (3.10)	***p* = 0.001**
Smoking					
Non smokers	30 (20.4%)	1.43 (0.83)	*F_W_*(2.82.91) = 9.44	0.34 (0.28)	*F_W_*(2.76.35) = 28.79
Ex-smokers	37 (25.2%)	1.76 (1.07)	***p* < 0.001**	0.63 (0.91)	***p* = 0.001**
Smokers	80 (54.4%)	2.44 (1.58)		2.11 (2.05)	
Age of smoking onset ^(†)^					
≤ 18 years old	45 (56.3%)	2.57 (1.76)	*t*(78) = 0.83	2.08 (2.14)	*t*(78) = −0.16
> 18 years old	35 (43.7%)	2.27 (1.32)	*p* = 0.410	2.15 (1.96)	*p* = 0.876
Number of cigarettes smoked per day ^(†)^					
< 20 cigarettes/day	30 (37.5%)	1.87 (1.01)	*t*(78) = −2.60	1.31 (1.22)	*t*(78) = −2.81
≥ 20 cigarettes/day	50 (62.5%)	2.78 (1.76)	***p* = 0.011**	2.58 (2.30)	***p* = 0.006**
Pack-year categories ^(††)^					
Non smokers	67 (45.6%)	1.61 (0.98)	*F_W_*(3.41.19) = 7.19	0.50 (0.71)	*F_W_*(3.38.14) = 17.53
Light smokers	26 (17.7%)	1.67 (0.80)	***p* = 0.001**	1.10 (0.87)	***p* < 0.001**
Moderate smokers	41 (27.9%)	2.67 (1.46)		2.36 (1.74)	
Heavy smokers	13 (8.8%)	3.25 (2.39)		3.32 (3.46)	

Mean, average number of participants; SD, Standard deviation; df, degrees of freedom.

(*) Independent samples *t*-test, One way Analysis of Variance–ANOVA (F), Welch’s ANOVA–(F_W_).

(†) Data refer to self-reported smokers only.

(††) Pack-years = (Number of cigarettes smoked per day/20) × Years of smoking.

Non-smokers = 0 pack years, Light- smokers = 1–20 pack years, Moderate smokers = 20–40 pack years, Heavy- smokers ≥ 40 pack years.

Bold Values indicate statistical significance.

### 3.6 Bivariate analyses between blood lead-cadmium levels and working characteristics – parameters

To assess the relationship between blood lead and cadmium concentrations and occupational characteristics, bivariate analyses were conducted. The analyses revealed statistically significant differences in the mean levels of both lead [*F_W_*(2.69.35) = 7.24, *p* = 0.001] and cadmium [*F_W_*(2.86.18) = 7.67, *p* = 0.001] across the three occupational specialty groups. Post-hoc comparisons indicated that workers in the high-exposure group had significantly higher mean blood lead concentrations (2.69 μg/dL) than general labor and maintenance workers (1.75 μg/dL, *p* = 0.002) and supervisory or administrative personnel (1.67 μg/dL, *p* = 0.005). Similarly, cadmium levels were significantly higher in the high-exposure group (2.12 μg/L) compared with general workers (1.07 μg/L, *p* = 0.016) and supervisory or administrative staff (0.73 μg/L, *p* = 0.001).

**Figure 1:**
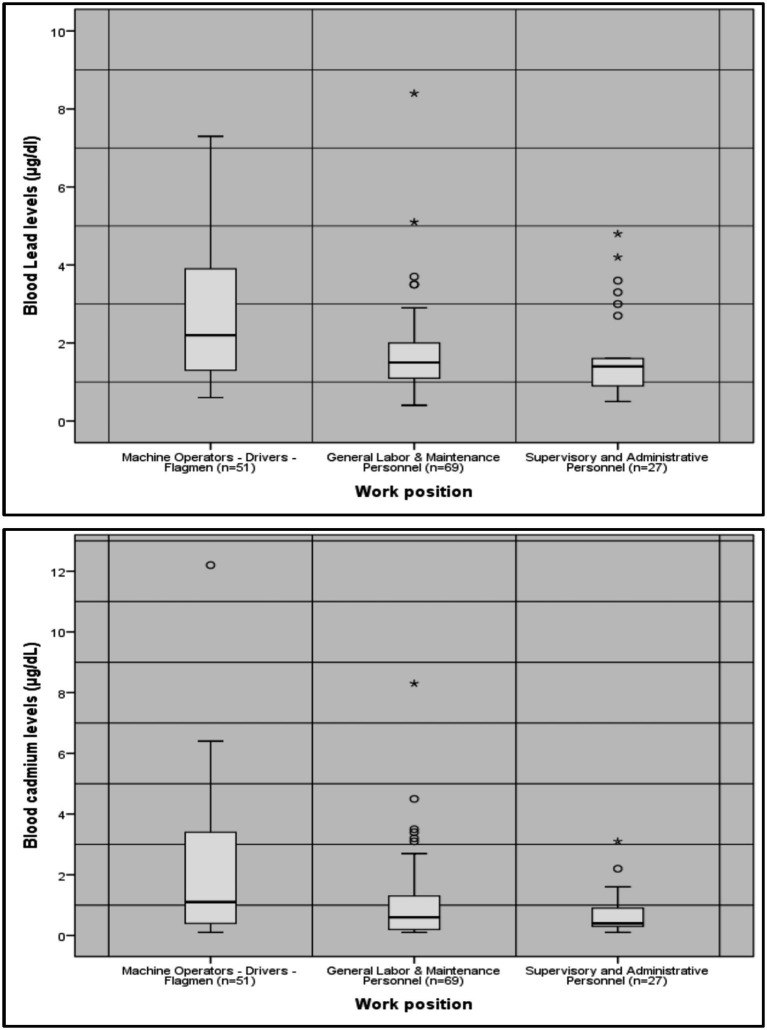
Distribution of blood lead (μg/dL) and cadmium (μg/L) concentrations across work positions, categorized according to exposure intensity. Boxplots represent median values, interquartile ranges, and overall variability, illustrating differences in internal metal burden by work-related exposure level.

Figure 1 illustrates the differences in metal concentrations across work positions, reflecting varying levels of occupational exposure.

Significant differences were also observed in lead and cadmium concentrations among employees working at different facilities [lead: *F_W_*(3.49.13) = 11.12, *p* < 0,001; cadmium: *F_W_*(3.44.44) = 11.95, *p* < 0.001]. *Post hoc* analysis indicated that workers employed at active landfill sites had significantly higher blood lead levels (2.57 μg/dL) compared with those working at recycling management centers (1.73 μg/dL, *p* = 0.034), inactive landfills (1.36 μg/dL, *p* < 0.001), and environmental parks (1.59 μg/dL, *p* = 0.002). Regarding cadmium, workers at inactive landfill sites had significantly lower blood concentrations (0.47 μg/L) compared with those employed at active landfills (1.73 μg/L, *p* < 0.001) and recycling centers (1.89 μg/L, *p* = 0.033).

In addition, duration of employment was significantly associated with both lead and cadmium concentrations [lead: *F_W_*(2.92.25) = 3.12, *p* = 0.049; cadmium: *F*_W_(2.81.37) = 3.88, *p* = 0.025]. Workers with 10–19 years of employment exhibited significantly higher mean blood lead levels (2.48 μg/dL) compared with those with less than 10 years of service (1.80 μg/dL, *p* = 0.049). A similar trend was observed for cadmium, with higher concentrations among workers employed for 10–19 years than among those with less than 10 years of employment (*p* = 0.020).

Detailed results of the bivariate analyses examining associations between lead and cadmium levels and occupational characteristics are presented in Table 5.

**Table 5:** Blood lead and cadmium levels according to occupational characteristics and work-related parameters of the participants.

Characteristic–parameter	Total (*Ν* = 147)	Blood lead level (μg/dL)	Test statistic^*^–*p*-value	Blood cadmium level (μg/L)	Test statistic^*^ *p*-value
	*n* (%)	Mean (SD)		Mean (SD)	
Work position					
Machine operators–drivers–flagmen	51 (34.7%)	2.69 (1.61)	*F_W_*(2.69.35) = 7.24	2.12 (2.38)	*F_W_*(2.86.18) = 7.67
General labor and maintenance personnel	69 (46.9%)	1.75 (1.15)	***p* = 0.001**	1.07 (1.31)	***p* = 0.001**
Supervisory and administrative personnel	27 (18.4%)	1.67 (1.15)		0.73 (0.71)	
Occupational site					
Active landfill site	76 (51.7%)	2.57 (1.66)	*F_W_*(3.49.13) = 11.12	1.73 (2.07)	*F_W_*(3.44.44) = 11.95
Recycling material management center	19 (12.9%)	1.73 (0.99)	***p* < 0.001**	1.89 (2.02)	***p* < 0.001**
Inactive landfill site	33 (22.4%)	1.36 (0.46)		0.47 (0.40)	
Environmental parks (former landfill areas)	19 (12.9%)	1.59 (0.76)		1.02 (0.94)	
Years of employment					
< 10 years	58 (39.5%)	1.80 (1.23)	*F_W_*(2.92.25) = 3.12	1.00 (1.03)	*F_W_*(2.81.37) = 3.88
10–19 years	53 (36.1%)	2.48 (1.70)	***p* = 0.049**	2.01 (2.47)	***p* = 0.025**
≥ 20 years	36 (24.5%)	1.86 (0.97)		1.04 (1.19)	

Mean, average number of participants; SD, Standard deviation; df, degrees of freedom.

(*) independent samples *t*-test, Welch’s ANOVA–(F_W_).

Bold Values indicate statistical significance.

### 3.7 Predictors of lead and cadmium levels. Hierarchical linear regression analysis

Hierarchical linear regression analyses were performed to identify predictors of blood lead and cadmium concentrations. The primary aim was to determine whether workplace characteristics and exposure level remained independent predictors of heavy metal levels after adjusting for demographic factors (age and years of employment) and behavioral confounders (smoking and alcohol use). Sex was excluded from the regression models because the flagmen–driver group consisted exclusively of men; including this variable resulted in substantial collinearity and biased estimates.

### 3.8 Lead levels

In the first step of the hierarchical regression analysis (Block 1), demographic variables (age and years of employment) were entered into the model. These factors explained 8.3% of the variability in blood lead levels, and the model was statistically significant [*F*(2,144) = 6.54, *p* = 0.002]. At this stage, only age was significantly and positively associated with blood lead levels (*B* = 0.054 μg/dL, *p* = *p* = 0.001), while years of employment were not.

In the second step (Block 2), behavioral factors—smoking exposure (pack-years) and alcohol abuse—were added. Their inclusion substantially improved the model, increasing the explained variance by 17.1% (Δ*R*^2^ = 0.171, *F*(2, 142) = 16.33, *p* < 0.001). In this model, higher blood lead levels were significantly associated with older age (*B* = 0.042 μg/dL, *p* = 0.005), greater smoking exposure (*B* = 0.025 μg/dL per pack-year, *p* < 0.001), and alcohol abuse (*B* = 0.931 μg/dL, *p* = 0.014).

In the final step (Block 3), workplace category was added, with operators–drivers–flagmen used as the reference group. This further improved the model, explaining an additional 6.9% of the variance (Δ*R*^2^ = 0.069, *F*(2, 140) = 7.13, *p* = 0.001), bringing the total explained variance to 32.4%. These results indicate that job position is an independent predictor of blood lead levels, even after controlling for demographic and behavioral factors. Compared with operators–drivers–flagmen, general workers had significantly lower blood lead levels (*B* = −0.819 μg/dL, *p* < 0.001), as did administrative and supervisory staff.

Age, smoking, and alcohol abuse remained significant predictors in the final model. Each additional year of age was associated with an increase of 0.041 μg/dL in blood lead levels (*p* = 0.005). Smoking exposure showed a clear dose–response relationship. More specifically, every 10 pack per year increase, corresponds to an increase of approximately 0.24 μg/dL in blood lead. For example, a heavy smoker (>40 pack-years) would be expected to have blood lead levels at least 0.96 μg/dL higher than a non-smoker, regardless of job position. Alcohol consumption was also associated with higher lead levels, with affected workers showing an average increase of 0.807 μg/dL (*p* = 0.027) (see Table 6).

**Table 6:** Hierarchical regression coefficients for predicting blood lead levels (μg/dL) among the participants (*Ν* = 147).

Variable (Predictor)	*Β*	95% CI (Β)	*SE*	*β*	*t*	*p*-value
Block 1						
Age (years)	0.054	(0.213, 0.085)	0.016	0.293	3.44	***p* = 0.001**
Years of employment	−0.003	(−0.037, 0.032)	0.015	−0.014	−0.16	*p* = 0.869
*R*^2^ = 0 0.083, *F*(2, 144) = 6.54, *p* = 0.002						
Block 2						
Age (years)	0.042	(0.013, 0.070)	0.014	0.226	2.89	***p* = 0.005**
Years of employment	−0.011	(−0.042, 0.021)	0.016	−0.053	−0.68	*p* = 0.498
Smoking (pack years)	0.025	(0.013, 0.037)	0.006	0.326	4.23	***p* < 0.001**
Alcohol use (ref. category: No)	0.931	(0.192, 1.670)	0.374	0.190	2.49	***p* = 0.014**
*R*^2^ = 0 0.255, Δ*R*^2^ = 0.171, *F*(2, 142) = 16.33, *p* < 0.001						
Block 3						
Age (years)	0.041	(0.013, 0.068)	0.014	0.220	2.88	***p* = 0.005**
Years of employment	−0.020	(−0.050, 0.011)	0.015	−0.097	−1.28	***p* =** 0.202
Smoking (pack years)	0.024	(0.013, 0.035)	0.006	0.312	4.22	***p* < 0.001**
Alcohol use (ref. category: No)	0.807	(0.094, 1.520)	0.361	0.165	2.24	***p* = 0.027**
Work position:(*) general labor and maintenance personnel	−0.819	(−1.256, −0.382)	0.221	−0.294	−3.70	***p* < 0.001**
Work position:(*) supervisory and administrative personnel	−0.681	(−1.254, −0.109)	0.290	−0.190	−2.35	***p* = 0.020**
*R*^2^ = 0 0.324, Δ*R*^2^ = 0.069, *F*(2, 140) = 7.13, *p* = 0.001						

CI, Confidence Interval; SE, Standard Error.

Pack-years = (Number of cigarettes smoked per day/20) × Years of smoking.

(*) Reference category for the variable of work position is Machine operators–Drivers–Flagmen.

*F*_block1_ (2,144) = 6, 54, *p* = 0.002, *F*_block2_ (4,142) = 12, 13, *p* < 0.001, *F*_block3_ (6, 140) = 11, 16, *p* < 0.001 (The reported *p*-values refer to the statistical significance of each corresponding model (block) in the hierarchical regression analysis).

Bold Values indicate statistical significance.

### 3.9 Cadmium levels

In the first step of the hierarchical regression analysis (Block 1), age and years of employment were entered into the model. These variables explained only 1% of the variance in blood cadmium levels (*R*^2^ = 0.01), and the model was not statistically significant (*p* = 0.473). Neither age (*B* = 0.017 μg/L, *p* = 0.423) nor duration of employment (*B* = 0.014, *p* = 0.556) was independently associated with cadmium levels at this stage.

In the second step (Block 2), behavioral factors—smoking exposure (pack-years) and alcohol abuse—were added to the model. This resulted in a substantial and statistically significant improvement (Δ*R*^2^ = 0.256, *F*(2, 142) = 24.72, *p* < 0.001), with the model explaining 26.6% of the variance in blood cadmium levels. Among these variables, smoking exposure was the only significant predictor, showing a positive association with cadmium concentrations (*B* = 0.046 μg/L, *p* < 0.001), indicating that greater cumulative smoking was linked to higher blood cadmium levels. Alcohol abuse showed a tendency toward higher cadmium levels but did not reach statistical significance (*B* = 0.779 μg/L, *p* = 0.101).

In the final step (Block 3), job category was included to assess its effect after controlling for demographic and behavioral factors. The final model explained 33.0% of the variance in blood cadmium levels and represented a significant improvement over the previous model (Δ*R*^2^ = 0.064, *F*(2,140) = 6.69, *p* = 0.002). Smoking exposure remained a strong predictor, with each additional pack-year associated with an increase of 0.044 μg/L in blood cadmium levels (*p* < 0.001). Job category also emerged as an independent predictor: compared with operators–drivers–flagmen (reference group), administrative and supervisory staff had significantly lower cadmium levels (*B* = −1.135 μg/L, *p* = 0.002), as did general workers in tasks and workshops (*B* = −0.881 μg/L, *p* = 0.002).

Alcohol consumption was not significantly associated with cadmium levels in the final model (*B* = 0.660 μg/L, *p* = 0.150), and age and years of employment remained non-significant. Overall, these findings indicate that, independent of age, work duration, and smoking behavior, workplace characteristics play an important role in predicting blood cadmium levels (see Table 7).

**Table 7:** Hierarchical regression coefficients for predicting blood cadmium levels (μg/L) among the participants (*Ν* = 147).

Variable (Predictor)	*Β*	95% CI (*Β*)	SE	*β*	*t*	*p*-value
Block 1						
Age (years)	0.017	(−0.024. 0.058)	0.021	0.071	0.80	*p* = 0.423
Years of employment	0.014	(−0.032. 0.059)	0.023	0.052	0.59	*p* = 0.556
*R*^2^ = 0.010, *F*(2, 144) = 0.75, *p* = 0.473						
Block 2						
Age (years)	−0.002	(−0.038. 0.034)	0.018	−0.010	−0.12	*p* = 0.900
Years of employment	0.001	(−0.038. 0.041)	0.020	0.006	0.07	*p* = 0.942
Smoking (pack years)	0.046	(0.031. 0.060)	0.007	0.465	6.09	***p* < 0.001**
Alcohol use (ref. category: No)	0.779	(−0.154. 1.711)	0.472	0.125	1.65	*p* **=** 0.101
*R*^2^ = 0.266 Δ*R*^2^ = 0.256, *F*(2, 142) = 24.72, *p* < 0.001						
Block 3						
Age (years)	−0.007	(−0.043, 0.028)	0.018	−0.032	−0.42	*p* = 0.675
Years of employment	−0.009	(−0.048, 0.029)	0.020	−0.036	−0.48	*p* = 0.632
Smoking (pack years)	0.044	(0.030, 0.058)	0.007	0.448	6.08	***p* < 0.001**
Alcohol use (ref. category: No)	0.660	(−0.242, 1.562)	0.456	0.106	1.45	***p* =** 0.150
Work position:(*) General labor and maintenance personnel	−0.881	(−1.434, −0.028)	0.280	−0.248	−3.15	***p* = 0.002**
Work position:(*) Supervisory and administrative personnel	−1.135	(−1.859, −0.410)	0.367	−0.248	−3.10	***p* = 0.002**
*R*^2^ = 0 0.330, Δ*R*^2^ = 0.064, *F*(2, 140) = 6.69, *p* = 0.002						

CI, Confidence Interval; SE, Standard Error.

Pack-years = (Number of cigarettes smoked per day/20) × Years of smoking.

(*) Reference category for the variable of work position is Machine operators–Drivers–Flagmen.

*F_block1_* (2, 144) = 0.75, *p* = 0.473, *F*_block2_ (4, 142) = 12, 86, *p* < 0.001, *F*_block3_ (6,140) = 11, 49, *p* < 0.001 (The reported *p*-values refer to the statistical significance of each corresponding model (block) in the hierarchical regression analysis).

Bold Values indicate statistical significance.

## 4 Discussion

The present study provides evidence of occupational exposure to lead and cadmium among sanitary landfill workers in Northern Greece, with exposure levels varying significantly according to job position, workplace site, and duration of employment. Workers in high-exposure roles—machine operators, drivers, and flagmen—exhibited significantly higher blood concentrations of both metals compared with moderately and low-exposed personnel. These findings support the hypothesis that direct contact with waste, leachate, dust, and emissions in active landfill environments constitutes a considerable route of heavy metal exposure. Although measured concentrations remained below established occupational safety limits, the observed gradients across exposure groups indicate that routine landfill operations may contribute to chronic low-level metal accumulation.

The observed blood lead and cadmium levels in this study were generally below current European Union (EU) occupational biological limit values, as well as reference levels reported by the Centers for Disease Control and Prevention (CDC) and the World Health Organization (WHO) for the general population, indicating relatively low exposure in this cohort. These findings are also consistent with previously reported ranges from European biomonitoring studies, suggesting comparable exposure levels among waste management workers in similar occupational settings. Lead is a non-essential, cumulative toxic metal with no known safe exposure level; the CDC considers blood lead levels ≥3.5 μg/dL as elevated, while the WHO emphasizes that even low-level exposure may result in adverse health effects (19, 20). In occupational settings, the EU has established a biological limit value (BLV) of 30 μg/dL during a transitional period, to be reduced to 15 μg/dL, alongside mandatory medical surveillance (21). Similarly, cadmium is a cumulative toxicant, with blood levels in the general population typically below approximately 1–2 μg/L in non-smokers and higher in smokers due to tobacco exposure, while occupational exposure is generally expected to remain below 5 μg/L (22, 23).

Behavioral factors emerged as strong and consistent predictors of heavy metal burden, particularly smoking (24, 25). A clear dose–response relationship was observed between smoking intensity (pack-years and cigarettes per day) and both lead and cadmium levels, with smoking remaining the most influential predictor of cadmium concentrations in multivariable analyses. This finding is consistent with previous research identifying tobacco smoke as a major non-occupational source of cadmium and lead exposure (24). Alcohol consumption was also associated with elevated lead levels, even after adjustment for confounders, suggesting a potential synergistic effect between lifestyle factors and occupational exposure (26, 27). These results highlight the importance of considering behavioral risk factors when interpreting occupational exposure data and designing preventive strategies.

Occupational characteristics retained independent predictive value in hierarchical regression models, underscoring the role of workplace-related exposure beyond individual behaviors. Job position remained a significant predictor of both lead and cadmium levels after adjustment for age, employment duration, smoking, and alcohol abuse, indicating that work tasks and proximity to waste handling activities are critical determinants of exposure. Additionally, employees working at active landfill sites exhibited higher metal concentrations than those employed at inactive landfills or environmental parks, suggesting that ongoing waste deposition and processing amplify exposure risks. The association between intermediate employment duration (10–19 years) and higher metal levels may reflect cumulative exposure before potential job reassignment or health-related work modifications among longer-tenured employees.

Hematological, biochemical, and blood pressure parameters were evaluated to investigate potential subclinical effects of chronic exposure to lead and cadmium, given their known impact on hematopoietic, renal, and cardiovascular systems. In the present study, no clinically meaningful differences were observed between occupational groups, suggesting that exposure levels were not sufficient to induce detectable systemic effects. This finding is consistent with the relatively low blood concentrations observed and may also reflect the implementation of systematic occupational health surveillance programs, including periodic medical examinations and biomonitoring, allowing early detection and prevention of adverse effects. Additionally, the absence of overt clinical manifestations may be partly explained by the “healthy worker effect,” whereby employed populations generally exhibit better health status than the general population, potentially leading to an underestimation of exposure-related morbidity.

Despite these findings, several limitations should be considered. The cross-sectional design precludes causal inference and does not allow assessment of temporal changes in exposure or health outcomes. In addition, metal concentrations in blood primarily reflect recent exposure and may underestimate long-term body burden, particularly for lead, which accumulates in bone, and for cadmium, which has been associated with reduced bone mineral density and osteoporosis (15, 24, 28). Furthermore, reliance on self-reported data for behavioral factors and PPE use may introduce reporting bias. It should be noted that PPE use is mandated by law; therefore, it was consistently reported by participants, limiting the ability to assess differences in adherence. The questionnaire used was not formally validated, which may affect the reliability of self-reported information. Moreover, environmental measurements (e.g., air, soil, or dust concentrations of metals) were not available, restricting the ability to directly link internal exposure levels with external environmental conditions. Potential confounding by lifestyle factors, particularly smoking—a known source of cadmium exposure—cannot be fully excluded, although it was considered in the analysis. Finally, the sample size, although adequate for the primary analyses, limited the number of variables that could be included in multivariable models, potentially constraining the exploration of additional associations.

Despite these limitations, this study provides novel biomonitoring data in a European context where comparable research is scarce. The findings contribute to a better understanding of occupational exposure patterns in landfill workers and highlight the importance of continued occupational health surveillance, consistent use of personal protective equipment, and integrated interventions targeting both workplace exposures and modifiable lifestyle risk factors.

## Data Availability

The raw data supporting the conclusions of this article will be made available by the authors, without undue reservation.

## References

[1] Abidin A. U. Maziya F. B. Susetyo S. H. Yoneda M., & Matsui Y. (2024). Heavy metal air pollution in an Indonesian landfill site: characterization, sources, and health risk assessment for informal workers. Environ Adv 15:100512. doi:10.1016/j.envadv.2024.100512

[2] Chaudhary R Nain P Kumar A. Temporal variation of leachate pollution index of Indian landfill sites and associated human health risk. Environ Sci Pollut Res. (2021) 28:28391–406. doi: 10.1007/s11356-021-12383-1, 33543433

[3] Essien J. P. Ikpe D. I. Inam E. D. Okon A. O. Ebong G. A., & Benson N. U. (2022). Occurrence and spatial distribution of heavy metals in landfill leachates and impacted freshwater ecosystem: an environmental and human health threat. PLoS One 17:e0263279. doi:10.1371/journal.pone.0263279, 35113945 PMC8812908

[4] Karimian S Shekoohiyan S Moussavi G. Health and ecological risk assessment and simulation of heavy metal-contaminated soil of Tehran landfill. RSC Adv. (2021) 11:8080–95. doi: 10.1039/d0ra08833a, 35423317 PMC8695097

[5] Kasassi A Rakimbei P Karagiannidis A Zabaniotou A Tsiouvaras K Nastis A . Soil contamination by heavy metals: measurements from a closed unlined landfill. Bioresour Technol. (2008) 99:8578–84. doi: 10.1016/j.biortech.2008.04.010, 18508262

[6] Podlasek A Vaverková MD Jakimiuk A Koda E. A comprehensive investigation of geoenvironmental pollution and health effects from municipal solid waste landfills. Environ Geochem Health. (2024) 46:97. doi: 10.1007/s10653-024-01852-4, 38393507 PMC10891210

[7] Ghobakhloo S Mostafaii GR Khoshakhlagh AH Moda HM Gruszecka-Kosowska A. Health risk assessment of heavy metals in exposed workers of municipal waste recycling facility in Iran. Chemosphere. (2024) 346:140627. doi: 10.1016/j.chemosphere.2023.140627, 37944764

[8] Julander A Lundgren L Skare L Grandér M Palm B Vahter M . Formal recycling of e-waste leads to increased exposure to toxic metals: an occupational exposure study from Sweden. Environ Int. (2014) 73:243–51. doi: 10.1016/j.envint.2014.07.006, 25300751

[9] Decharat S. Heavy metals exposure and hygienic behaviors of workers in sanitary landfill areas in southern Thailand. Scientifica. (2016) 2016:9269210. doi: 10.1155/2016/9269210, 27313961 PMC4893594

[10] Al-Khashman OA. Heavy metal distribution in dust, street dust and soils from the work place in Karak industrial estate. Jordan Atmos Environ. (2004) 38:6803–12. doi: 10.1016/j.atmosenv.2004.09.011

[11] Chen TB Zheng YM Lei M Huang ZC Wu HT . Assessment of heavy metal pollution in surface soils of urban parks in Beijing. China Chemosphere. (2005) 60:542–51. doi: 10.1016/j.chemosphere.2004.12.072, 15950046

[12] Tüzen M. Determination of heavy metals in soil, mushroom and plant samples by atomic absorption spectrometry. Microchem J. (2003) 74:289–97. doi: 10.1016/S0026-265X(03)00035-3

[13] Chalvatzaki E Aleksandropoulou V Lazaridis M. A case study of landfill workers exposure and dose to particulate matter-bound metals. Water Air Soil Pollut. (2014) 225:1782. doi: 10.1007/s11270-013-1782-z

[14] Huang G Xie J Li T Zhang P. Worker health risk of heavy metals in pellets of recycled plastic: a skin exposure model. Int Arch Occup Environ Health. (2021) 94:1581–9. doi: 10.1007/s00420-021-01727-6, 34283290

[15] Taha MM Mahdy-Abdallah H Shahy EM Ibrahim KS Elserougy S. Impact of occupational cadmium exposure on bone in sewage workers. Int J Occup Environ Health. (2018) 24:101–8. doi: 10.1080/10773525.2018.1518745, 30222069 PMC6237150

[16] Bashir B Jarial S Sood S Vishwas S. Heavy metals and Alzheimer’s disease: a review. Heavy Metal Toxicity and Neurodegeneration. (2025) 225-230. doi: 10.1016/B978-0-443-36575-1.00020-0

[17] Schwartz J. The relationship between blood lead and blood pressure in the NHANES II survey. Environ Health Perspect. (1988) 78:15–22. doi: 10.1289/ehp.887815, 3203634 PMC1474612

[18] Wang Y Xu T Zhang Y He Y Fang J Xu Y . Interaction between depression and non-essential heavy metals (cd, Pb, and hg) on metabolic diseases. J Trace Elem Med Biol. (2024) 85:127484. doi: 10.1016/j.jtemb.2024.127484, 38924924

[19] Centers for Disease Control and Prevention (CDC). Blood Lead Level Guidance. Atlanta: CDC (2024). Available online at: https://www.cdc.gov/niosh/lead/bll-reference/index.html (Accessed April 2, 2026)

[20] World Health Organization. Lead Poisoning and Health. Geneva: WHO (2023). Available online at: https://www.who.int/news-room/fact-sheets/detail/lead-poisoning-and-health (Accessed April 2, 2026)

[21] European Parliament and Council. Directive (EU) 2024/869 of the European Parliament and of the council on the protection of workers from the risks related to exposure to lead and diisocyanates at work. (2024). Available online at: https://eur-lex.europa.eu/legal-content/EN/TXT/HTML/?uri=OJ:L_202400869 (Accessed April 2, 2026)

[22] Agency for Toxic Substances and Disease Registry (ATSDR). Toxicological Profile for Cadmium. Atlanta (GA): U.S. Department of Health and Human Services, Public Health Service (2012). Available online at: https://www.atsdr.cdc.gov/toxprofiles/tp5.pdf (Accessed April 2, 2026)

[23] World Health Organization. Cadmium in Drinking-water. Background Document for Development of WHO Guidelines for Drinking-water Quality. Geneva: WHO (2011). Available online at: https://cdn.who.int/media/docs/default-source/wash-documents/wash-chemicals/cadmium.pdf (Accessed April 2, 2026)

[24] Bernhard D. Rossmann A., & Wick G. (2005). Metals in cigarette smoke. IUBMB Life 57.: 805–809. doi:10.1080/15216540500459667 16393783

[25] Genchi G Sinicropi MS Lauria G Carocci A Catalano A. The effects of cadmium toxicity. Int J Environ Res Public Health. (2020) 17:3782. doi: 10.3390/ijerph17113782, 32466586 PMC7312803

[26] Shin J Luo Y. Relationship between exposure to multiple heavy metals and depressive symptoms in the US: the impact of alcohol consumption. Heliyon. (2024) 10:e40221. doi: 10.1016/j.heliyon.2024.e40221, 39669164 PMC11635719

[27] Yang AM Hu XB Liu S Cheng N Zhang DS Li JS . Occupational exposure to heavy metals, alcohol intake, and risk of type 2 diabetes and prediabetes among Chinese male workers. Chronic Dis Transl Med. (2019) 5:97–104. doi: 10.1016/j.cdtm.2019.05.002, 31367698 PMC6656874

[28] Alfvén T Järup L Elinder CG. Cadmium and lead in blood in relation to low bone mineral density and tubular proteinuria. Environ Health Perspect. (2002) 110:699–702. doi: 10.1289/ehp.110-1240916, 12117647 PMC1240916

[29] Li B Deng J Li Z Chen J Zhan F He Y . Contamination and health risk assessment of heavy metals in soil and ditch sediments in long-term mine wastes area. Toxics. (2022) 10:607. doi: 10.3390/toxics10100607, 36287888 PMC9610562

